# Efficient Spin Injection into Silicon and the Role of the Schottky Barrier

**DOI:** 10.1038/srep03196

**Published:** 2013-11-12

**Authors:** André Dankert, Ravi S. Dulal, Saroj P. Dash

**Affiliations:** 1 Department of Microtechnology and Nanoscience, Chalmers University of Technology, SE-41296, Göteborg, Sweden

## Abstract

Implementing spin functionalities in Si, and understanding the fundamental processes of spin injection and detection, are the main challenges in spintronics. Here we demonstrate large spin polarizations at room temperature, 34% in n-type and 10% in p-type degenerate Si bands, using a narrow Schottky and a SiO_2_ tunnel barrier in a direct tunneling regime. Furthermore, by increasing the width of the Schottky barrier in non-degenerate p-type Si, we observed a systematic sign reversal of the Hanle signal in the low bias regime. This dramatic change in the spin injection and detection processes with increased Schottky barrier resistance may be due to a decoupling of the spins in the interface states from the bulk band of Si, yielding a transition from a direct to a localized state assisted tunneling. Our study provides a deeper insight into the spin transport phenomenon, which should be considered for electrical spin injection into any semiconductor.

Spintronics exploits the spin of the electron, rather than its charge, for information storage and processing^1^,^2^,^3^. Developing methods for efficient injection, controlled manipulation, and sensitive detection of electron spins in semiconductors has the potential to profoundly affect information technology. The strong interest in silicon spintronics rises from the expected long spin coherence length and its industrial dominance^4^. Creating spin polarized carriers in Si by using polarized light^5^, hot electrons spin injection^6^, tunnel spin injection^7^,^8^,^9^,^10^,^11^,^12^,^13^,^14^,^15^, Seebeck spin tunneling^16^, and dynamical spin pumping methods has been demonstrated recently^17^. The use of ferromagnetic tunnel contacts to inject and detect spin polarizations in Si has been recognized as the most viable and robust method among them^4^,^18^. Recently, optical detection of spin polarization, through analysis of the degree of polarization of the light emitted by spin–LED structures, showed 30% spin polarization in Si at 77 K^8^,^9^. In comparison, an all-electrical nonlocal measurement method that represents the detection of spin accumulation in the Si gives rise to a spin polarization of less than 1%^10^,^11^. More recently, it has become possible to probe large spin accumulations directly underneath the injection contact up to room temperature by means of the Hanle effect, using a three-terminal configuration^4^,^7^,^12^,^13^. In these experiments spin-polarization values of 5% in n-type Si at 300 K have been observed by using Al_2_O_3_, MgO, plasma oxidized SiO_2_, and graphene tunnel barriers together with ferromagnetic contacts^4^,^7^,^12^,^13^,^19^. These low values of spin-polarization obtained through electrical methods are mainly due to the lack of high-quality tunnel barriers on Si, which prevents efficient spin injection and detection. The challenge is to achieve spin polarization in Si close to the tunnel spin-polarization values of ferromagnetic contacts.

Another important issue is the mechanism of spin injection and detection in semiconductors. The semiconductor/tunnel-barrier/ferromagnet contacts are associated with a Schottky barrier and carrier depletion at the semiconductor surface. This gives rise to different spin-transport processes, depending on the profile of the Schottky barrier^20^,^21^. It has been proposed that, for degenerate semiconductors, a very narrow Schottky barrier would allow direct spin-polarized tunneling, while in nondegenerate semiconductors, the presence of a wider Schottky barrier would be expected to change the transport mechanism^20^. In the latter case, the depletion region is too wide for tunneling and thermionic and localized state-assisted transport are expected to dominate. It is proposed that such transport processes give rise to anomalous behavior in spin accumulation and detection^20^,^21^,^22^,^23^. Although anomalous changes in the sign of spin signals have been observed in different semiconductors^24^,^25^,^26^, experiments to elucidate this effect have not yet been performed.

In this article, we demonstrate the creation of large spin polarizations in n-type and p-type Si, using ozone oxidized SiO_2_ as a tunnel barrier, and we address the role played by the profile of the Schottky barrier in the processes of spin injection and detection. Degenerate Si provides a very narrow Schottky barrier, which allows efficient spin injection and detection in the direct-tunneling regime over a wide temperature range. Furthermore, increasing the width of the Schottky barrier in nondegenerate Si results in an anomalous sign change of the spin signal in the low bias regime. This can be due to the change in transport processes across the interface from direct to indirect tunneling, since spins accumulated in localized states can be decoupled from the Si bands by the Schottky barrier. Our observations are generic in nature, and are also valid for different tunnel barrier materials on Si.

## Results

### Large electron spin polarization in degenerate n-type Silicon

**Figure 1:**
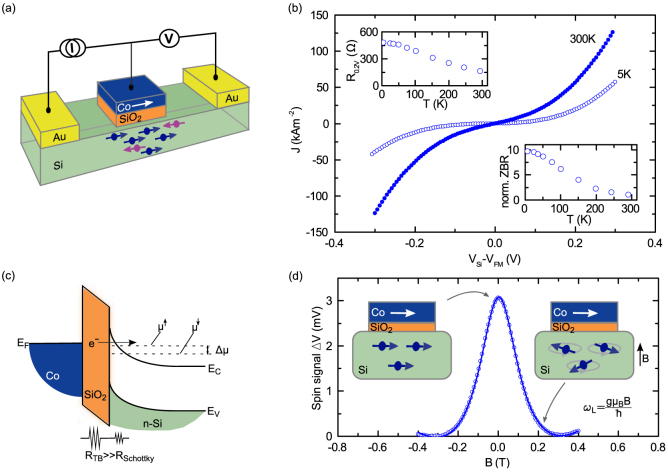
Large spin signal in degenerate n-type Si. (a) Three-terminal device geometry for injection and detection of spin polarization in Si with SiO_2_/Co tunnel contacts. (b) Current density versus bias voltage of the n++ Si/SiO_2_/Co tunnel contact measured in a three-terminal geometry at different temperatures. Insets: Temperature dependence of junction resistance at bias voltages of zero and +200 mV. (c) Energy-band diagram showing the injection of spin-polarized current through a ferromagnetic tunnel contact into n-type Si, creating a majority spin accumulation and spin-splitting of electrochemical potential in the conduction band. (d) Electrical detection of large spin polarization in n-type Si at 300 K through the Hanle effect. The spin signal of 3 mV is observed for +1 V bias voltage and +34 mA bias current. The solid line is a Lorentzian fit with a lower limit for the spin lifetime *τ_eff_* = 50 ps. Left panel: The maximum spin accumulation in the absence of external magnetic field B. Right panel: A finite perpendicular magnetic field causes spin precession at the Larmor frequency, and results in the suppression of the spin accumulation.

To demonstrate large spin accumulations by direct tunneling, we used SiO_2_/Co tunnel contacts on degenerate n-type Si (n++ Si; measured electron density *n* = 3 · 10^19^ cm^−3^ at 300 K). The SiO_2_ barrier was prepared by ozone oxidation (see Methods). Electrical measurements were performed in a three-terminal geometry (Fig. 1a), in which the same tunnel interface is used for injection and for detection of spin accumulation in Si^7^,^22^,^27^.

The contacts show the tunneling behavior characterized by nonlinear, quasisymmetric *J*–*V* characteristics with a weak temperature dependence (Fig. 1b)^29^. We obtain a resistance-area-product in the junction of *R_junc_A* ~ 4 Ωmm^2^ at room temperature, which increases only by factor two when cooling down to 5 K. Additional to the low temperature dependence, we observed an exponential dependence of junction resistance with SiO_2_ tunnel barrier thickness (supplementary Fig. S1). These results indicate the growth of an uniform and pinhole free SiO_2_ tunnel barrier on Si. Such barriers were used in ferromagnetic tunnel contacts of Co/SiO_2_ to create an majority spin accumulation and splitting of electrochemical potential in the conduction band of n++ Si (Fig. 1c). The Hanle effect is used to control the reduction of the induced spin accumulation by applying an external magnetic field (*B*) perpendicular to the carrier spins in the Si. The spin accumulation decays as a function of *B* with an approximately Lorentzian line shape given by 
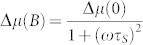
, where Δ*μ*(0) and Δ*μ*(*B*) are respectively the spin accumulation in zero magnetic field and in a finite perpendicular magnetic field, and *τ_S_* is the spin lifetime^7^. Figure 1d shows the measurement of a large electrical Hanle signal, Δ*V* = 3 mV, for a Co/SiO_2_/n++ Si junction at 300 K and for a constant tunnel current of *I* = 34 mA at a bias voltage of 1 V. In the linear response regime, this large signal corresponds to a spin splitting of 
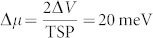
 (with assumed SiO_2_/Co contact tunnel spin polarization TSP = 0.35). The Fermi–Dirac distributions for the majority (*n*^↑^) and minority (*n*^↓^) electron spin densities are found to be 2 · 10^19^ cm^−3^ and 10^19^ cm^−3^, respectively^4^,^7^. This corresponds to a giant electron spin polarization of 
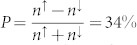
 in the n++ Si conduction band, which is nearly one order of magnitude greater than previously reported values^4^,^7^,^12^,^13^,^19^ and close to the TSP of the ferromagnetic tunnel contact at room temperature. The low-defect density of the ozone-oxidized SiO_2_ tunnel barrier and its better interface quality with Si offers a great advantage, suggesting a means to produce a large spin polarization in Si at room temperature.

**Figure 2:**
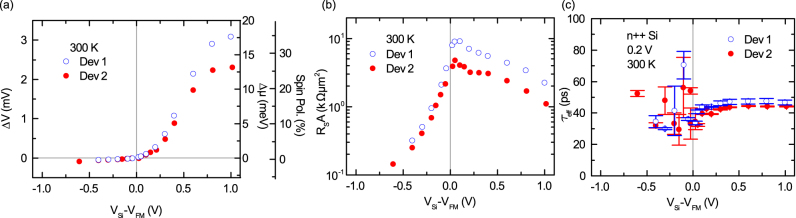
Bias dependence of the spin signal for degenerate n-type Si. Measurements are shown for two different devices. (a) The bias dependence of the measured Hanle voltage signal (Δ*V*), the calculated spin splitting (Δ*μ*), and the spin polarization (*P*) created in the Si conduction band due to spin injection at 300 K. The bias voltages *V_Si_* − *V_FM_* > 0 and *V_Si_* − *V_FM_* < 0 correspond to spin injection and spin extraction, respectively. (b) Bias dependence of spin-RA product (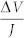) at 300 K. (c) The bias dependence of the effective spin lifetime (*τ_eff_*) at 300 K with error bars.

Investigating the bias voltage and temperature dependence of the Hanle spin signal provides more detailed quantitative information about spin-tunnel processes. The bias dependence of the Hanle spin signal (Δ*V*), the calculated spin splitting and spin polarization are presented in Fig. 2a. For *V_Si_* − *V_FM_* > 0 (reverse bias), the spin injection from the SiO_2_/Co tunnel contact creates a majority spin accumulation, while for *V_Si_* − *V_FM_* < 0 (forward bias), the spin extraction creates a minority spin accumulation in the Si. The magnitude of the spin accumulation varies asymmetrically with the bias voltage across the junction, even though the current density is almost symmetric with respect to bias-voltage polarity. The measured Hanle signal for the majority and minority spin accumulation are opposite in sign. For spin injection, the signal increases linearly with low bias current, and saturates at higher bias (see supplementary Fig. S10 for bias current dependence). The Hanle curves at different bias voltages are presented in the supplementary information (supplementary Fig. S2).

**Figure 3:**
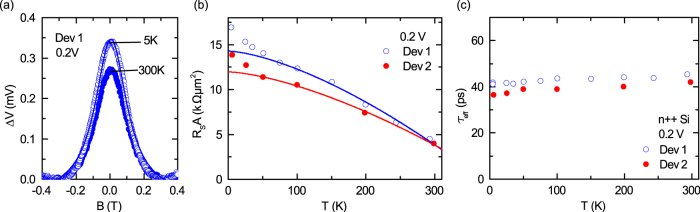
Temperature dependence of the spin signal for degenerate n-type Si. Measurements are shown for two different devices. (a) Hanle spin signal at 5 K and 300 K for an applied bias voltage of +0.2 V (b) Temperature dependence of spin-RA at an applied bias voltage of +0.2 V. The lines represent the theoretical prediction for spin accumulations created through direct tunneling: 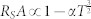 28. (c) Temperature dependence of the effective spin lifetime (error bars are smaller than data points).

The spin-resistance-area product (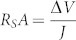) is found to be in the range 1–4 kΩ*μ*m^2^ (Fig. 2b), which is large, compared to theoretical predictions^4^,^30^. In the diffusive regime, *R_S_A* should be equal to *P*^2^*ρ_Si_L_sd_* = 10 Ω*μ*m^2^, where *ρ_Si_* = 3 mΩcm at 300 K, and *L_sd_* is the spin diffusion length^4^. Although the experimental values are large, we can rule out any enhancement of the spin signal by tunneling through localized states over the full temperature range. The weak temperature dependence of the Hanle spin signal (Fig. 3a) and *R_S_A* (Fig. 3b) at low bias voltages matches the theoretical predictions 
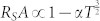
 7,28. This indicates a true spin accumulation in the Si conduction band over the full temperature range, since localized interface states are expected to provide a larger temperature dependence of *R_S_A*^22^,^31^. The thinner, low resistance Schottky barrier of this highly doped n++ Si devices couples well the localized states and the Si conduction band allowing for direct spin-polarized tunneling^14^,^23^. It should also be noted that the weak temperature dependence of SiO_2_ has an advantage over other oxide tunnel barriers such as Al_2_O_3_^4^ and MgO^13^,^31^, in which *R_S_A* increases exponentially at lower temperatures. Such low temperature dependence of spin signal has also been reported using plasma SiO_2_ tunnel barriers on Si^12^. From the separately measured diffusion constant 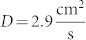 and the Lorentzian fitting of the Hanle curves, the lower limit for the spin lifetime and the spin diffusion length are found to be 
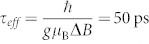
 and 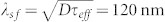, respectively, at room temperature. These values match very well previously reports for such highly doped Si at room temperature^7^,^18^,^32^. Furthermore, the spin lifetime is found to be independent of both temperature and bias voltage (Fig. 2c and 3c), supporting the direct tunneling and detection of spin accumulations in the bulk Si conduction band over the measured temperature and bias voltage ranges.

### Large hole spin polarization in degenerate p-type Silicon

**Figure 4:**
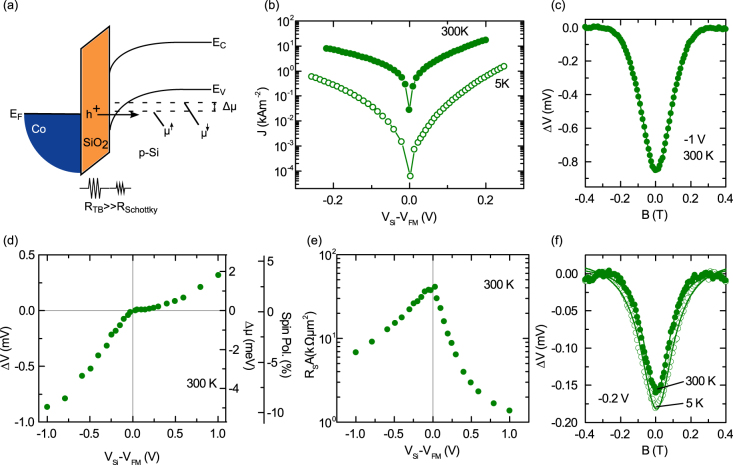
Spin signal for degenerate p-type Si. (a) Energy-band diagram for SiO_2_/Co tunnel contact with p++ Si. The injection of spin-polarized holes creates a spin accumulation and spin-splitting of electrochemical potential in the Si valence band. (b) Current density–bias voltage characteristics at 5 and 300 K. The bias voltages, *V_Si_* − *V_FM_* < 0 and *V_Si_* − *V_FM_* > 0, correspond to spin injection and spin extraction, respectively. (c) Large Hanle signal measured for spin-polarized hole injection at −1 V bias voltage and −3.17 mA bias current at 300 K. (d) Bias dependence of Hanle spin signal at 300 K, calculated spin splitting and spin polarization created in the Si valence band. (e) Bias dependence of *R_S_A* at 300 K. (f) Temperature dependence of Hanle spin signal measured at 5 and 300 K at a bias voltage of −0.2 V.

By employing similarly fabricated SiO_2_/Co tunnel contacts on degenerate p-type Si (p++ Si, hole density *p* = 1.8 · 10^19^ cm^−3^ at 300 K), we have studied spin-polarized hole accumulations through direct tunneling. Figure 4a shows the energy-band diagram and the creation of spin splitting in the valence band of p++ Si due to electrical spin injection. The *J*–*V* characteristics of the tunnel junction at 5 K and 300 K are shown in Fig. 4b. Hanle measurements at room temperature demonstrate a large spin signal of −0.87 mV at −1 V bias voltage and −3.17 mA bias current (Fig. 4c), which corresponds to a spin splitting in the p++ Si valence band of 
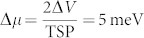
, and majority (*p*^↑^) and minority (*p*^↓^) hole spin densities of 0.99 · 10^19^ cm^−3^ and 0.81 · 10^19^ cm^−3^, respectively. This indicates that hole spin polarization is large, at 
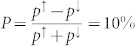
. It should be noted that we have not taken into consideration the valence-band spin splitting that would be present in p-type Si due to the spin-orbit interaction.

The bias dependence of the Hanle spin signal is shown in Fig. 4d. Spin injection (*V_Si_* − *V_FM_* < 0) and spin extraction (*V_Si_* − *V_FM_* > 0) produce a net excess of holes, with majority and minority spin, respectively. As expected, a change in the sign of the Hanle signal is observed, corresponding to these majority and minority spin accumulations. The Hanle signals are presented in supplementary Fig. S3, as measured at different bias voltages. The *R_S_A* shows asymmetric behavior, decreasing more quickly for the case of spin extraction than in the case of spin injection (Fig. 4e). Although the *R_S_A* values observed are larger than expected on the basis of the spin-diffusion model^4^,^22^,^30^, the narrow Schottky barrier for p++ Si should rule out spin accumulation in localized states^31^. This is supported by the very weak temperature dependence of the Hanle signal observed at a low bias voltage of −200 mV, providing a clear indication of the true spin accumulation in the Si valence band (Fig. 4f). From the Lorentzian fitting of the Hanle curves, the lower limit for the spin lifetime is found to be *τ_S_* ~ 50 ps, and spin diffusion length is seen to be *L_SD_* ~ 80 nm. These values are similar to those from previous reports on degenerate p-type Si as measured by electrical spin injection^7^, thermal spin injection^16^, and spin pumping^17^. However, understanding the large spin lifetimes observed in p-type Si at room temperature remains an open question^33^.

### Spin injection into nondegenerate Si and anomalous spin signals

**Figure 5:**
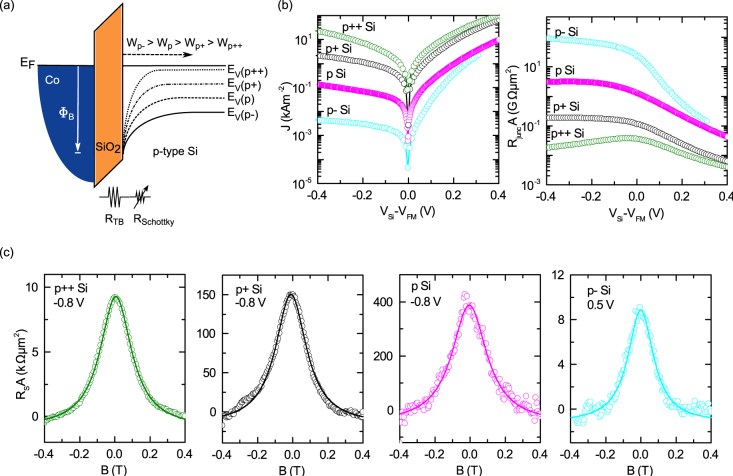
Tailored Schottky barrier width and Hanle spin signals for p-type Si. Si devices with four different boron doping concentrations were studied. (a) Energy-band diagram for p-type Si/SiO_2_/Co showing doping-dependent Schottky barrier width. (b) Bias voltage dependence of current density and *R_junc_A* at 300 K. (c) Hanle spin signals measured at 300 K.

In the previous sections, we have presented results on spin injection into degenerate Si with very narrow Schottky barriers, where the transport process is dominated by direct tunneling. However, in nondegenerate Si, the presence of a wider Schottky barrier alters the transport process. In order to determine the effects of the Schottky barrier profile on the spin injection and detection process, we perform experiments on Si with different doping densities, i.e. with different Schottky barrier widths *W* and hence resistances (see Fig. 5a). We use p-type Si with four different boron doping concentrations (see Table I for the parameters of the Si samples). The preparation conditions of the ozone-oxidized SiO_2_ tunnel barrier and ferromagnetic contacts are kept identical for all these devices. Figure 5b shows the significant decrease in current density or increase in resistance in the reverse bias regime upon lowering the boron doping density, showing the transition of the contacts from tunneling to diodic behavior. Such systematic variation of the Schottky barrier resistance is quite useful in verifying the proposed spin-transport models.

Hanle spin signals with a Lorentzian line shape have been successfully measured for all four p-type Si samples at room temperature (Fig. 5c). The magnitude of the spin signal is found to increase with decreasing doping density or with increasing resistance of the Schottky barrier. Such scaling of the spin signal has been recently demonstrated with tunnel barrier resistance^34^. We do not observe a change in the effective spin life time with decreasing doping concentration in silicon, which is in agreement with the lower limit for the derived spin lifetime *τ_eff_*^32^.

**Figure 6:**
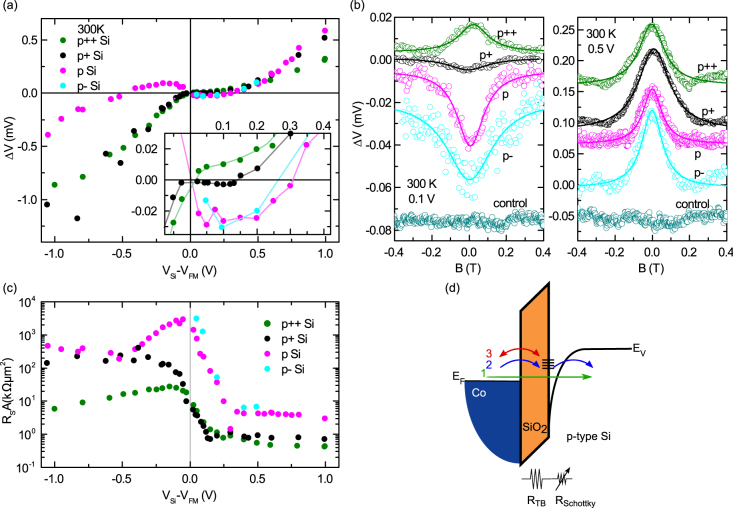
Bias dependence of spin signal with tailored Schottky barrier width at room temperature. (a) Bias dependence of Hanle spin signals for four different boron doping concentrations in p-type Si. The degenerate p++ Si device shows normal Hanle signal behavior, whereas the nondegenerate devices (p+, p and p- Si) show anomalous sign reversal. The inset shows the low bias regime, in order to emphasize the sign reversal of the spin signals. (b) Hanle curves at 100 mV (left panel) and 500 mV (right panel) demonstrating the sign reversal exists only in the low bias regime. A control sample with nonmagnetic layer between FM and tunnel barrier demonstrates clearly the origin of the spin signal in the Si band. (c) *R_S_A* with bias voltage for four devices with different boron doping concentrations in Si. (d) Energy-band diagram showing different possible spin-transport mechanisms across the tunnel junction, depending on the resistance of the tunnel and Schottky barrier. For a fixed SiO_2_ tunnel-barrier resistance, a low Schottky barrier resistance leads primarily to direct tunneling between the ferromagnet and the Si (1). With increasing Schottky barrier resistance, the two-step tunneling into Si via localized states (2), and the tunneling between the ferromagnet and the localized states at the interface (3), become more dominant.

Studying the bias dependence of the Hanle spin signals is an excellent way to investigate the role of the Schottky barrier, as the applied bias voltage defines the energy profile at which both spin injection and detection take place. Figure 6a shows this bias dependence of the Hanle signals on the different boron doped Si devices. With decreasing doping concentration, the width of the Schottky barrier increased, leading to increased resistance, which in turn yielded an unusual sign change in the Hanle signal at low bias voltages.

For nondegenerate p+ Si, an unusual sign reversal in the spin signal is detected in the spin extraction regime (0 < *V_Si_* − *V_FM_* < 140 mV). By further decreasing the doping concentration (p Si), the sign reversal of the Hanle signal becomes more prominent, that is, it increases in magnitude and extends to the higher bias regime of 400 mV. Interestingly, a sign change is also observed in the spin injection regime up to bias voltages of −600 mV. Finally, for the lowest doped p- Si, the sign-reversal phenomenon is also observed in the spin extraction regime. In this case, the spin injection signal in the reverse bias condition could not be measured, as the higher resistance of the Schottky barrier decreased the signal-to-noise ratio. It should be noted that the sign reversal behavior of the Hanle signal is observed only at lower bias voltages; at higher bias voltages, the expected sign of the spin signal is restored. This is demonstrated exemplary in Fig. 6b for all four different p-type Si devices at two different bias voltages: At a low bias voltage of 0.1 V a clear spin signal sign change was observed, whereas at a higher bias voltage of 0.5 V regular Hanle signals without any sign inversion could be obtained. The detailed measurement of Hanle signals at different bias voltages, showing sign change for different p-type Si devices, are presented in supplementary Fig. S3 to Fig. S6. The sign reversal of the Hanle spin signal is also reproduced for different SiO_2_ tunnel barrier thicknesses and with an Al_2_O_3_ tunnel barrier (supplementary Fig. S7 and Fig. S8). To confirm the spin-polarized tunneling as origin of the Hanle signals, a control sample, prepared with a nonmagnetic interlayer (10 nm Ti) between the Co and the SiO_2_ tunnel barrier, is measured resulting in no spin signal (Fig. 6b and supplementary Fig. S10)^35^. The *R_S_A* values measured on the four doping concentrations are shown in Fig. 6c. In addition to the asymmetric behavior of the signal, a peak also appears at low bias voltages for p Si and p- Si devices. This enhancement of the spin signal specifically occurs in the bias voltage range where a sign inversion in the Hanle signal has been observed.

## Discussion

In this report we have presented results of large spin accumulations in degenerate Si by using an ozone oxidized SiO_2_ tunnel barrier. Narrow Schottky barriers in case of highly doped Si and an improved interface quality of the SiO_2_ tunnel barrier allows dominant direct tunneling of spin polarized electrons. The observed large magnitude of the spin accumulation can not be explained by the standard spin diffusion model^30^. Such large spin *R_S_A* has also been observed in literature and reviewed recently in detail^4^,^18^. Previously, various control experiments could rule out any enhancement of the spin signal at room temperature^7^,^20^, whereas a strong enhancement at low temperature was attributed to spin accumulations in localized states^22^,^31^. Here, we observe a very low temperature dependence with SiO_2_ tunnel barrier which rules out any signal enhancement over the range from 5–300 K. This is further supported by the absence of any variation of spin lifetime with temperature and bias voltage^22^,^31^. This demonstrates that high quality interfaces made of ozone oxidized SiO_2_ on degenerate Si, for a narrow Schottky barrier, allows efficient spin injection by direct tunneling mechanism. Nevertheless, a unified theory to explain large spin accumulation observed in semiconductors is still missing.

Furthermore, our experiments systematically show that larger Schottky barrier resistances induce and enhance the sign inversion of the spin signal. Such wider Schottky barrier decouple the localized states at the interfaces from the silicon bulk bands^23^. The anomalous spin-signal signs observed in different bias regimes can be due to competing transport processes across the tunnel and Schottky barrier^23^. There are three main transport processes, which can contributing in the different regimes (Fig. 6d): (1) At low Schottky barrier resistance (degenerate Si), or at high bias voltages, direct tunneling dominates, yielding a normal sign for the Hanle signal. (2) Resonant tunneling via localized states at the interface can occur for higher Schottky barrier resistances and competes with direct tunneling. At lower bias voltages, different escape times for up- and down-spins can give rise to an opposite spin accumulation resulting in an inversion of the Hanle signal. (3) The tunneling between the ferromagnet and the localized states can be dominant when the Schottky barrier resistance is very high, or when the applied bias voltage is low.

Previously, several experimental observation of bias dependent sign inversions of the spin signal have been made in both nonlocal and three-terminal measurement geometries^24^,^25^,^26^. Such sign inversion can be due to: (a) The energy at which the injection and detection takes place can be different giving rise to different and even negative spin polarization values for injection and detection^36^. (b) Different escape times of the spin carriers from localized states can give rise to spin accumulation with opposite spin orientation^21^. (c) The presence of acceptor and donor states of paramagnetic centers at the interfaces. Those mechanisms combined with complicated transport processes across the tunnel and Schottky barrier could be responsible for the sign reversal of the spin signal. However, this behavior is not clearly understood for a three-terminal measurement, since the Hanle signal arises from the spin potential difference created by a spin polarized current through a single tunnel contact. Although, the exact origin of the sign inversion of the spin signal is not clear yet, our experiments demonstrate that an increased Schottky barrier resistance can cause these anomalous behavior at low bias voltages.

In conclusion, we have demonstrated a giant spin accumulation in highly doped Si at room temperature using ozone-oxidized SiO_2_/Co tunnel junctions. We achieved a spin polarization of 34% in n-type Si, and 10% in p-type Si. Temperature and bias dependence measurements indicate that the spin polarization created in highly doped Si is dominated by a direct tunneling mechanism. The obtained spin polarization in n-type Si is almost one order of magnitude larger than previously reported, and is close to the tunnel spin polarization of the ferromagnetic tunnel contacts. The low-resistivity and defect density of the ozone-oxidized SiO_2_ tunnel barrier offers a great advantage over other tunnel barriers, suggesting a new route to producing large spin polarization in Si at room temperature. Additionally, spintronic devices based on SiO_2_ tunnel barriers are compatible with and can easily be integrated into the present Si technology. Opportunities are growing with the availability of new Heusler alloy materials that possess larger spin polarizations, and which can also be integrated with Si^37^. Furthermore, we address here the role of the Schottky barrier in spin injection and detection processes. Schottky barrier resistances above a critical limit result in a decoupling of spins in the localized states from the Si bulk bands resulting in an anomalous sign reversal in the spin signal in the lower bias voltage regime. This may be due to the domination of two-step tunneling and thermionic spin transport across the interface. These findings encourages further investigation to understand the effect of localized states and paramagnetic centers on the spin signal. This also requires generating an improved theoretical description of the spin injection and detection process. These results will enable utilization of the spin degree of freedom in complementary Si devices and its further development.

## Methods

### Device fabrication

SiO_2_ tunnel junction preparation: Spin-transport devices with ferromagnetic tunnel contacts of Co/SiO_2_ were fabricated on a number of Si substrates. The substrates were cleaned, and the native oxide was etched with diluted hydrofluoric acid. SiO_2_ tunnel oxide was formed by ozone oxidation at room temperature, with an O_2_ flow of (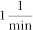) for 30 min. in the presence of a UV radiation source positioned 4 mm above the chips. The O_2_ molecules are broken down to atomic oxygen by the UV radiation and combine with O_2_ molecules to form ozone. Ozone oxidation is known to provide ultra-thin, uniform tunnel barriers, a fact also confirmed by the temperature and thickness dependence of our junction resistance measurements. The samples were then transferred to an electron beam deposition system, where 15 nm of ferromagnetic Co layer and 10 nm of Au capping layer were deposited. The ferromagnetic contacts were patterned by photolithography and Ar-ion beam etching. Tunnel contact areas of 200 × 100 *μ*m^2^ were used for measurements, allowing a sufficiently good signal-to-noise ratio. The Cr (10 nm)/Au (100 nm) reference contacts on the Si, and contact pads on the ferromagnetic tunnel contacts were prepared with the use of photolithography and the lift-off method. The reference Cr/Au contacts were used to source a DC current and to detect a voltage signal with respect to the ferromagnetic tunnel contact.

### Measurements

The measurements were performed in three-terminal geometry, with the spin polarization injected and detected using the same Si/SiO_2_/Co interface (Fig. 1a). A constant DC current was sent through this ferromagnetic tunnel interface into the Si, and the resulting voltage detected using a nanovoltmeter. The SiO_2_ tunnel resistance is much higher than that of the Co/Au metal electrodes and the degenerate Si; for nondegenerate Si, however, the Schottky barrier resistance dominates and determines the voltage drop. Measurements were performed in a variable temperature cryostat (5 K–300 K) with a superconducting magnet. Initially, a sufficiently large in-plane magnetic field (parallel to the tunnel interface) was applied to create a homogeneous in-plane magnetization of the ferromagnetic electrode. The spins injected into the Si by the source current were thus also oriented along the same axis. In order to measure the resulting spin accumulation in Si, we employed the Hanle effect, in which a small out-of-plane magnetic field is applied perpendicular to the spin direction. This has no significant effect on the orientation of the magnetization of the ferromagnetic electrode, but it does lead to precession of the spins in the Si, and thereby a controlled suppression of the spin accumulation. This changes the tunnel resistance of the active contact, which in turn produces a change in the detected voltage proportional to the spin accumulation.

## Author Contributions

A.D. fabricated and measured most of the devices. S.P.D. and R.S.D. contributed in some fabrication and measurements. S.P.D. and A.D. conceived the ideas and designed the experiments. S.P.D. planned and supervised the research. All authors contributed in the discussion and analysis of the measurements. S.P.D. and A.D. wrote the manuscript with input from R.S.D.

## Supplementary Material

Supplementary Information Supplementary Information

## Figures and Tables

**Table 1:** Parameters for degenerate n-type Si and the four p-type Si samples studied

Si type	Dopant	Doping density	Resistivity	Mobility	Schottky barrier width
		(cm^−3^)	(Ωcm)	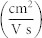	(nm)
n++	As	3 · 10^19^	0.003	118	3
p++	B	1.8 · 10^19^	0.005	51	7
p+	B	1.5 · 10^19^	0.008	57	8.3
p	B	5.4 · 10^18^	0.011	109	13.5
p−	B	1.3 · 10^15^	10	466	736
