# Supplementary material for: Efficient Spin Injection into Silicon and the Role of the Schottky Barrier
Source: Sci Rep. 2013 Nov 12;3:3196. doi: 10.1038/srep03196 (PMC3824168; doi:10.1038/srep03196)
Supplement: Supplementary Information [file srep03196-s1.pdf]

Supplementary information

Efficient Spin Injection into Silicon and the Role of the Schottky Barrier

André Dankert,* Ravi S. Dulal, and Saroj P. Dash†
Chalmers University of Technology,
Department of Microtechnology and Nanoscience,
Quantum Device Laboratory; Göteborg, Sweden

S1. SILICON DIOXIDE TUNNEL BARRIER GROWTH AND CHARACTERIZATION

The SiO_2 growth rate was calibrated on additional Si chips fabricated along with the chips for the ferromagnetic tunnel devices. The latter were used to study the spin accumulation and the junction resistance presented in the main paper. Initially, the thermal SiO_2 on the chips for calibration was completely removed by buffered hydrofluoric acid just before the ozone oxidation. Then, the thickness of the oxide layer was measured with an ellipsometer (J.A. Woollam M2000). The resulting oxide thickness depending on the oxidation time is shown in Fig. S1a. The obtained data exhibit a logarithmic growth of the tunnel barrier up to an oxidation time of 2 hours. Furthermore, the junction resistance of our devices correlates linearly with the oxidation time, which means the resistance increases exponential with the oxide thickness. Additionally, the conductivity dependence on the bias voltage can be fitted with the Brinkman-Dynes-Rowell (BDR) approach for asymmetric barriers (Fig. S1b) [1]. The exponential dependence of the resistivity on the SiO_2 barrier thickness and the characteristic BDR tunneling fit in combination with the low temperature dependence (main paper Fig. 1b) are clear indications of the good quality of the tunnel barrier.

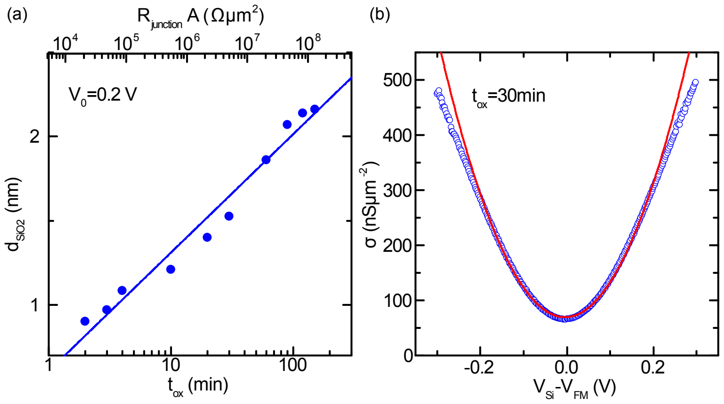

Figure S1: **Characterization of SiO2 tunnel barrier:** (a) SiO_2 thickness and junction resistance dependence on oxidation time. The resistance values are obtained from IV measurements at a bias voltage of 0.2 V, the thickness has been measured with an ellipsometer. (b) Bias dependence of the junction conductance fitted with Brinkman-Dynes-Rowell approach for asymmetric barriers [1].

This shows that our oxidation process with UV created ozone is able to create a uniform, pinhole free oxide layer up to 2.3 nm thickness, ranging the junction resistance-area product from $10^4 - 10^8\ \Omega\mu m^2$, which is excellent to study spin injection and detection in Si.

*Electronic address: andre.dankert@chalmers.se
†Electronic address: saroj.dash@chalmers.se

S2. DEGENERATE N-TYPE SILICON

Hanle measurements for n++ Si at several bias voltages in the injection ($V_{Si} - V_{FM} > 0$) and extraction ($V_{Si} - V_{FM} < 0$) regime are performed (Fig. S2a). Four typical Hanle curves, at low and high bias, are shown in Fig. S2b. Even for very low bias voltages, the spin-signal sign behaves normally, being positive for electron spin injection and negative for extraction.

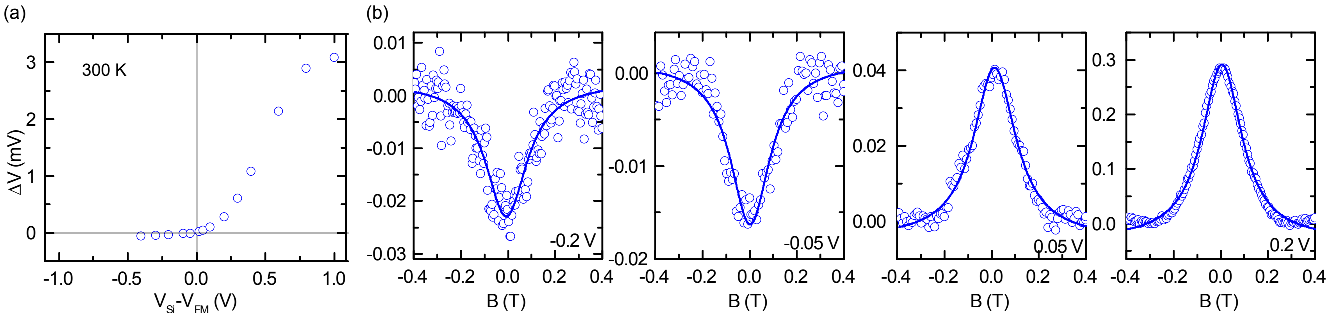

Figure S2: **Bias dependence of Hanle spin signal for n++ Si devices:** (a) Normal bias dependence for both spin injection and extraction at 300 K, without anomalous sign reversal of Hanle spin signal in the same bias regime (same as Fig. 2a). (b) Hanle curves for spin injection and extraction at ±50 mV and ±200 mV bias voltages.

S3. DEGENERATE AND NONDEGENERATE P-TYPE SI

On all p-type Si samples, the SiO_2 tunnel barrier was prepared identically to the n-type Si samples, as explained in the previous section and in the main paper. A 30 minute ozone treatment was also chosen for the four different boron doping concentrations compared in main Fig. 4 and Fig. 5. Figure S3 to Fig. S6 give an overview of the Hanle signal measurements. For p++ Si, the spin-signal sign behaves as expected, that is, negative for hole injection and positive for hole extraction (Fig. S3). Si with a higher Schottky barrier width (p+) exhibits a sign reversal for low bias hole extraction (Fig. S4). By further increasing the Schottky barrier width, and hence resistance (p Si), this sign reversal becomes more pronounced at higher bias voltages and in both the injection and extraction regimes (Fig. S5). For the lowest doping (p- Si) concentration, hole spin injection could not be observed, as the high Schottky barrier resistance decreases the signal-to-noise ratio, making measurements of Hanle signals impossible. Yet for hole extraction, a clear sign reversal can be observed (Fig. S6).

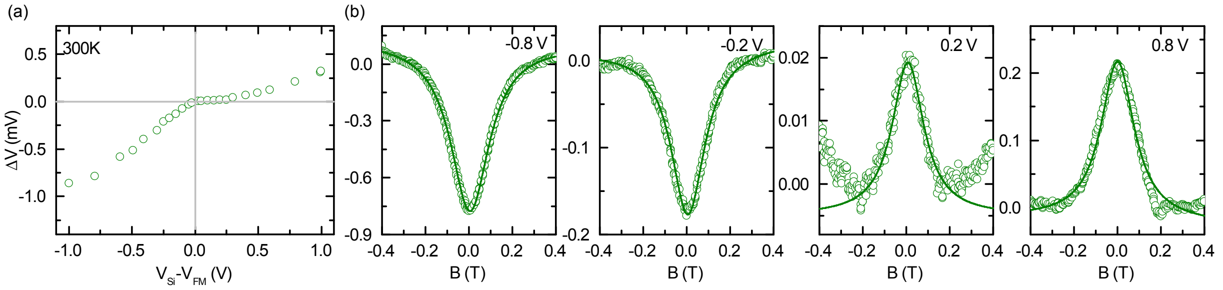

Figure S3: **Bias dependence of Hanle spin signal for the p++ Si device:** (a) Normal bias dependence for both spin injection and extraction at 300 K without anomalous sign reversal of the Hanle spin signal in the same bias regime (same as in main Fig. 3d). (b) Hanle curve for spin injection and extraction at ±0.2 V and ±0.8 V bias voltage.

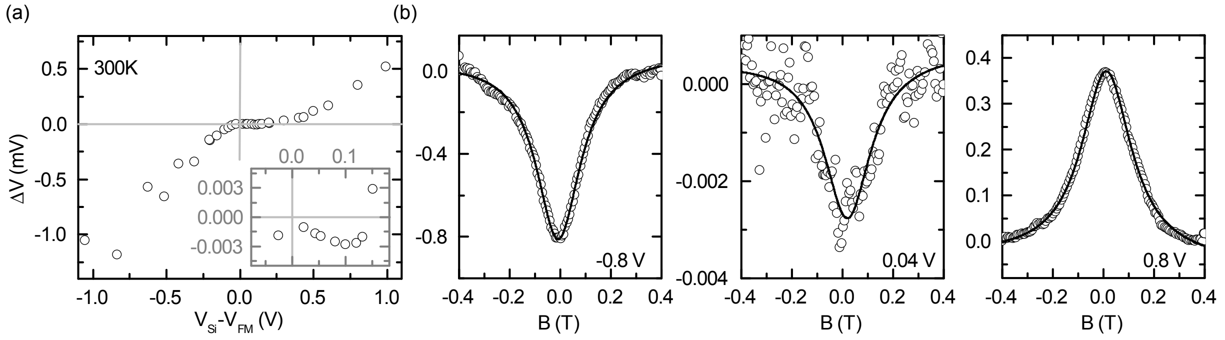

Figure S4: **Bias dependence of Hanle spin signal for the p+ Si device:** (a) Anomalous sign reversal of the Hanle spin signal at 300 K in the lower bias regime for spin extraction (same as Fig. 5a). Inset: magnification of low bias voltage regime to emphasize sign change. (b) Hanle curves for spin injection and extraction at bias voltages of ±0.8 V showing normal sign of the spin signal. However, the extraction signal at +0.04 V shows a sign reversal.

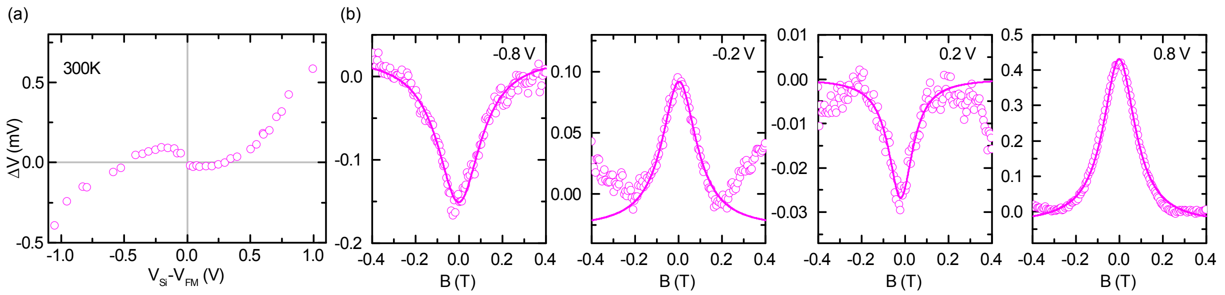

Figure S5: **Bias dependence of Hanle spin signal for the p Si device:** (a) Anomalous sign reversal of Hanle signal at 300 K for both injection and extraction in the lower bias regime. (b) Hanle measurements for spin injection and extraction at ±0.8 V bias voltage showing normal sign of spin signal. However, injection and extraction at ±0.2 V bias voltage show a sign reversal.

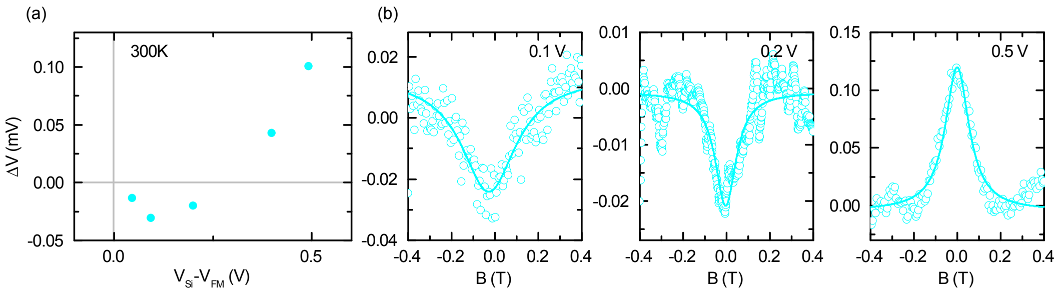

Figure S6: **Bias dependence of Hanle spin signal for the p- Si device:** (a) Anomalous sign reversal of Hanle signal for extraction in the lower bias regime. It should be noted that no Hanle signal could be measured for negative bias voltages, due to a decreased signal-to-noise ratio resulting from the wider Schottky barrier. (b) Hanle signal for spin extraction at 0.5 V showing normal spin signal, whereas at 0.1 V and 0.2 V a sign reversal occurs.

Furthermore, we also conducted experiments on p Si with different thickness of SiO_2 tunnel barrier, by reducing the ozone exposure time to 10 minutes, and on Al_2 O_3/Co tunnel contacts. The bias dependence shows a similar sign reversal as with the thicker SiO_2 tunnel barrier sample (Fig. S7 and Fig. S8). The Al_2O_3 sample was prepared identically to the n++ Si/Al_2O_3/Co device described in the previous section.

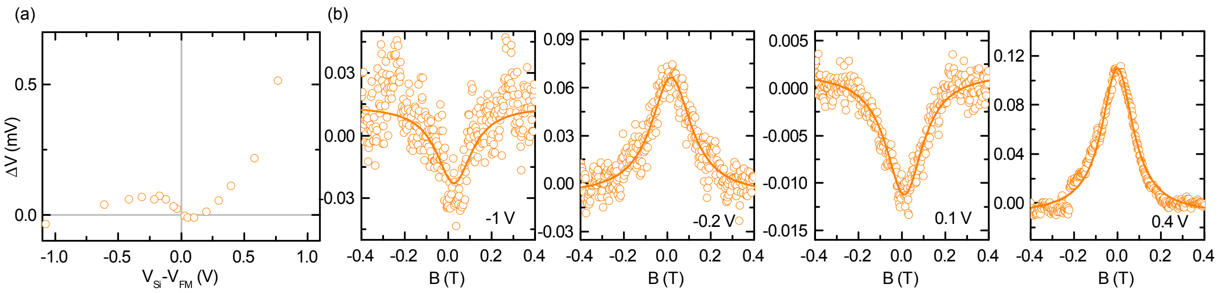

Figure S7: **Bias dependence of Hanle spin signal for p Si (with Ozone oxidation for 10 minutes)** (a) Anomalous sign reversal of Hanle signal at 300 K for both injection and extraction in the lower bias regime. (b) Hanle signal for spin injection and extraction at -1 V and $+0.4$ V bias voltage, respectively, show a normal spin signal. However, the spin signal at -0.2 V and $+0.1$ V show a sign reversal.

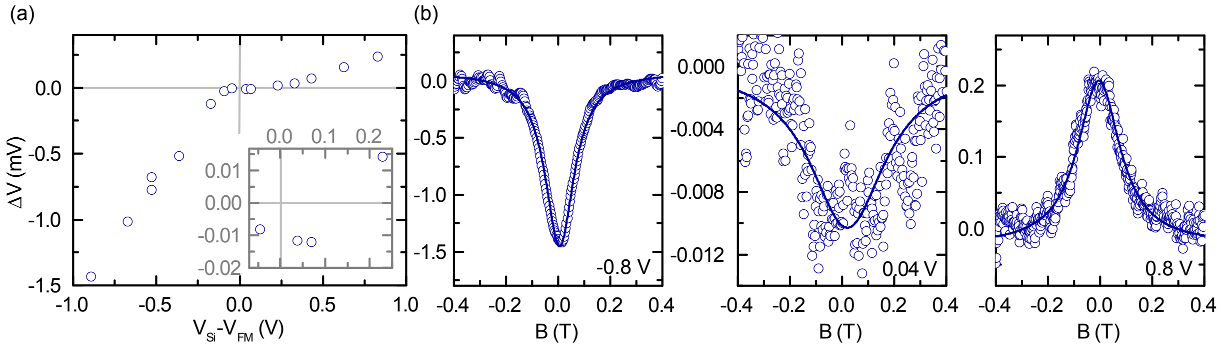

Figure S8: **Bias dependence of Hanle spin signal for p Si (with** 8 Å **evaporated Al_2O_3, followed by plasma oxidation)** (a) Anomalous spin sign reversal of the Hanle signal at 300 K only for spin extraction at lower bias voltages. Inset: magnification of low bias voltage regime to emphasize sign change. (b) Hanle curve for spin injection and extraction at ± 0.8 V bias showing normal sign of spin signal. However, the extraction signal at 0.04 V bias voltage shows a sign reversal.

S4. CONTROL EXPERIMENT

The resistance of the ferromagnetic contact and of the highly doped silicon are two orders of magnitude smaller than that of the tunnel barrier. However, the resistance of the Schottky barrier for low-doped silicon is much higher than the resistance of the tunnel-barrier. Hence, the resistances of the Schottky barrier also contribute to the measured voltage in low-doped Si devices. To rule out any unwanted contributions to the measured signal, we performed a decisive test using a control device with 10 nm of nonmagnetic Ti inserted between SiO_2 and Co in a p Si device. This is known to suppress the spin polarization of the injected tunnel current, resulting in zero spin accumulation [2]. We could not observe a Hanle signal for the Ti control devices over a broad range of bias voltage from -800 to 800 mV. Figure S9 compares Hanle measurements on the control sample with a regular p-type Si sample. This unambiguously demonstrates that the observed signals in our junctions represent spin accumulation induced by injection of a spin-polarized tunnel current.

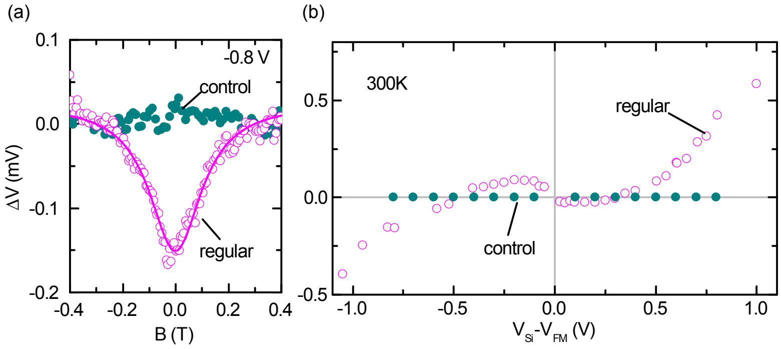

Figure S9: **Control measurement for p Si:** (a) Hanle measurement in control sample p Si/SiO_2/10 nm TiO_2/15 nm Co/10 nm Au compared with p Si/SiO_2/15 nm Co/10 nm Au at −0.8 V bias voltage. (b) Bias dependent measurement comparing FM tunnel contact (same as Fig. S5) with 10 nm Ti interlayer contact. As expected, no spin signal could be observed for the control sample over the range −0.8 V to 0.8 V.

S5. SPIN SIGNAL BIAS CURRENT DEPENDENCE

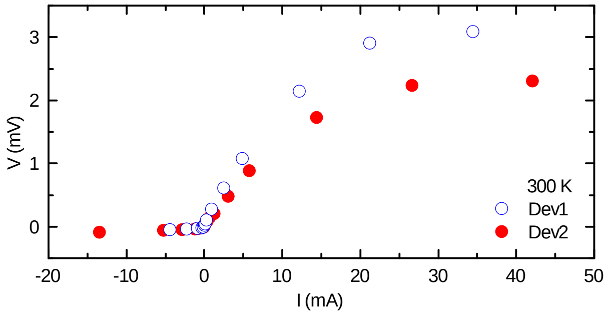

Figure S10: **Bias current dependence of degenerate n-doped Sample:** The spin signal data (identical to main Fig. 2a) depending on the bias current demonstrating a linear response for low bias currents and a saturation at higher bias.

[1] W. Brinkman *et al.* Tunneling conductance of asymmetrical barriers. *J. Appl. Phys.* **41** (1970).
[2] R. S. Patel *et al.* Magnetic tunnel contacts to silicon with low-work-function ytterbium nanolayers. *J. Appl. Phys.* **106**, 016107 (2009).
